# Supplementary material for: A geospatial examination of specialist care accessibility and impact on health outcomes for patients with acute traumatic spinal cord injury in New South Wales, Australia: a population record linkage study
Source: BMC Health Serv Res. 2021 Apr 1;21:292. doi: 10.1186/s12913-021-06235-4 (PMC8015029; doi:10.1186/s12913-021-06235-4)
Supplement: Supplementary file 1 — Additional file 1: Appendix 1. Datasets and variables used [file 12913_2021_6235_MOESM1_ESM.pdf]

APPENDIX 1: Datasets and variables used

Datasets

1. The **NSW Ambulance data collections** contain operational information from the Computer Aided Dispatch system, and clinician recorded data including vital signs, protocols applied and treatment/response within electronic medical records. NSW Ambulance datasets capture information for emergency and urgent episodes of care for patients who: were transported to a hospital by Ambulance, were left at a scene following clinician assessment, or died at the scene. It includes information on inter-hospital transfers, except for routine medical appointments. Variables contained within these data can be accessed at https://www.cherel.org.au/data-dictionaries#section1

2. The **NSW Admitted Patient Data Collection (APDC)** includes records for all hospital separations (discharges, transfers and deaths) from all NSW public and private hospitals and day procedure centres. The APDC records include a range of demographic data items (e.g. date of birth, residential address, language spoken at home and country of birth), administrative items (e.g. admission and separation dates) and coded information (e.g. reason for admission, significant co-morbidities and complications and procedures performed during the admission). Variables contained within these data can be accessed at https://www.cherel.org.au/data-dictionaries#section1

3. **NSW Activity Based Funding District Network Return (NSW-DNR)** is a state-based hospital cost data collection of individual patient cost and health service activity data. The NSW-DNR is prepared and submitted by each of the 15 Local Health Districts and three Specialty Health Networks (Districts/Networks). In NSW, financial results are published and audited at the District/Network level and not at hospital level. The NSW-DNR includes all products for all Districts/Networks and reconciles to the published financial results. NSW-DNRs are consolidated and formatted to comply with the National Hospital Costs Data Collection data set specifications.

A. TSCI IDENTIFICATION

The dataset combined all patients for whom one of the following ICD-10-AM (International Classification of Diseases – Tenth Revision, Australian Modification) codes have been identified in any separation, and in any position of the diagnostic code list within the NSW Admitted Patient Data Collection (APDC):

S12, S12.0, S12.1, S12.2, S12.21, S12.22, S12.23, S12.24, S12.25, S12.7, S12.8, S12.9, S13.1, S13.10, S13.11, S13.12, S13.13, S13.14, S13.15, S13.16, S13.17, S13.18, S13.2, S13.3, S14.0, S14.10, S14.11, S14.12, S14.13, S14.70, S14.71, S14.72, S14.73, S14.74, S14.75, S14.76, S14.77, S14.78, S22.0, S22.00, S22.01, S22.02, S22.03, S22.04, S22.05, S22.06, S22.1, S24.0, S24.1, S24.10, S24.11, S24.12, S24.7, S24.70, S24.71, S24.72, S24.73, S24.74, S24.75, S24.76, S24.77, S32, S32.0, S32.00, S32.01, S32.02, S32.03, S32.04, S32.05, S34.0, S34.1, S34.3, S34.70, S34.71, S34.72, S34.73, S34.74, S34.75, S34.76, T06.0, T06.1, T09.3.

B. Mechanism of injury and activity

Injury mechanism and activity at the time of injury were identified using the following ICD-10 AM codes

1. **Activity of injured person at time of injury**
   a. Sports and leisure: U50 – U72
   b. Other activity: U73
2. **Accidents**
   a. Transport accidents: V00 – V99
   b. Other external causes of accidental injury: W00 – X59
   c. Intentional self-harm: X60 – X84
   d. Assault: X85 – Y09
   e. Event of undetermined intention: Y20 – Y36
   f. Legal intervention and operations of war: Y35 – Y36

C. Multiple Trauma

Other injury categories in addition to TSCI were identified using the following ICD-10 AM codes. The other injury categories are:

a. Arm or Shoulder injury: S57, S58, S61, S62, S67, S68
b. Hip or Leg injury: S71, S72, S73, S77, S78, S79, S81, S82, S83, S87, S88, S91, S92, S93, S97, S98

c. Chest or Abdomen injury: S21, S22, S26, S27, S28, S28.0, S28.1, S31, S36, S38
d. Skull or Face injury: S01, S02, S05, S08 and
e. Traumatic Brain injury: S06, S07

D. Secondary Complications

The list of ICD-10 AM codes used to identify the secondary complications are given below;

List of Secondary Complications and ICD-10 AM Codes

COMPLICATION CATEGORY	ICD-10 AM CODE
**URINARY**	
**URINARY TRACT INFECTION**	N30, N39
**OTHER DISORDERS OF BLADDER**	N31
**URETHRITIS**	N34, N37
**HAEMATURIA**	R31
**OTHER URINARY COMPLICATION**	N99.8, N99.9
**URINARY RETENTION/INCONTINENCE**	R33, R32, N39.4, N39.3
**RESPIRATORY**	
**PNEUMONITIS DUE TO SOLIDS AND LIQUIDS**	J69
**PNEUMONIA, PULMONARY COLLAPSE**	J12, J14, J15, J17, J18, J98.1
**RESPIRATORY FAILURE**	J96.9, J96.0, J96.1
**PLEURISY**	R09.1, J86.9, J86.0, J90
**PRESSURE INJURIES**	
**DECUBITUS ULCERS (PRESSURE ULCER)**	L89

E. New South Wales Activity Based Funding District Network Return Data

Cost categories included within APDC and EDDC DNR data collections;

a. Employee-Related Costs,
b. Visiting Medical Officers,
c. Other Operating Costs,
d. Finance Costs,
e. Depreciation,

f. Amortisation,
g. Grants, and
h. Subsidies.
